# Colchicine Use and Risks of Stroke Recurrence in Acute Non-Cardiogenic Ischemic Stroke Patients: A Population-Based Cohort Study

**DOI:** 10.3390/jpm11090935

**Published:** 2021-09-19

**Authors:** Chi-Hung Liu, Yu-Sheng Lin, Pi-Shan Sung, Yi-Chia Wei, Ting-Yu Chang, Tsong-Hai Lee, Ching-Yu Lee, Yan-Rong Li

**Affiliations:** 1 Stroke Center and Department of Neurology, Linkou Chang Gung Memorial Hospital, Taoyuan 333, Taiwan; ivanliu0519@gmail.com (C.-H.L.); t_y_chang@hotmail.com (T.-Y.C.); thlee@cgmh.org.tw (T.-H.L.); 2 College of Medicine, Chang Gung University, Taoyuan 333, Taiwan; dissertlin@gmail.com; 3 Division of Cardiology, Department of Internal Medicine, Chiayi Chang Gung Memorial Hospital, Chiayi 613, Taiwan; 4 Department of Neurology, National Cheng Kung University Hospital, College of Medicine, National Cheng Kung University, Tainan 704, Taiwan; pishansung@gmail.com; 5 Department of Neurology, Keelung Chang Gung Memorial Hospital, Keelung 204, Taiwan; yichiawei@gmail.com; 6 Institute of Neuroscience, National Yang-Ming University, Taipei 112, Taiwan; 7 Department of Orthopaedics, Taipei Medical University, School of Medicine, College of Medicine, and Taipei Medical University Hospital, Taipei 110, Taiwan; ejaca22@gmail.com; 8 Division of Endocrinology and Metabolism, Department of Internal Medicine, Linkou Chang Gung Memorial Hospital and College of Medicine, Chang Gung University, No. 5, Fu-Hsing St., Kueishan, Taoyuan 333, Taiwan

**Keywords:** Asian, atrial fibrillation, colchicine, ischemic stroke, statin, prevention

## Abstract

**Background:** The objective is to study whether the cardiovascular protective effects of colchicines could be applied to non-cardiogenic ischemic stroke (IS) patients. **Patients and Methods:** Non-cardiogenic IS patients were identified from the National Health Insurance Research Database. Eligible patients were divided into chronic and non-chronic use categories based on their long-term status of colchicine use. The non-chronic use category was subdivided into (1) non-user and (2) new user groups while the chronic use category was divided into (3) former user and (4) long-term user groups according to the patient’s recent status of colchicine use. Inverse probability of treatment weights for propensity scores was used to balance the baseline characteristics. The primary outcome was recurrent IS, which was compared within the non-chronic use and chronic use categories. **Results:** In the non-chronic use category, the number of patients was 355,498 and 912 in the non-user and new user groups, respectively. In the chronic use category, the number of patients was 4737 and 4354 in the former user and long-term user groups, respectively. In the non-chronic use category, patients in the new user group had a marginally lower risk of recurrent IS at 6-months (subdistribution hazard ratio [SHR], 0.95; 95% confidence interval [CI], 0.94–0.97) and 2-years (SHR, 0.92; 95% CI, 0.91–0.93) follow up. In the chronic use category, patients in the long-term user group also had a marginally lower risk of recurrent IS at 6-months (SHR, 0.87; 95% CI, 0.86–0.88) and 2-years (SHR, 0.87; 95% CI, 0.86–0.88) follow up. The effect of colchicine on the reduced risk of recurrent IS was more favorable in patients who also used statins. **Conclusions:** Recent colchicine use in acute non-cardiogenic IS patients is associated with marginal fewer incidences of recurrent IS. Patients with concurrent statin use may have more profound protective effects.

## 1. Introduction

Risk factor modification and antithrombotic drugs are crucial for reducing ischemic stroke (IS) recurrence [1,2]. Recent data has indicated that atherosclerotic plaque inflammation may be an important contributor to plaque destabilization and thromboembolic events [3]. Therefore, an anti-inflammatory approach is evolving for the prevention of atherosclerotic cardiovascular (CV) disease [3]. Statins are a well-known approach with lipid lowering and anti-inflammatory effects [4]. Clinical trials have demonstrated the promising effects of high-potency atorvastatin on the prevention of secondary IS [5], and better protective effects of aggressive lipid lowering therapy on atherosclerotic stroke [6].

Colchicine is an anti-inflammatory treatment which attracted much clinical attention [4]. The Colchicine Cardiovascular Outcomes (COLCOT) and Low-Dose Colchicine (LoDoCo) trials have demonstrated that patients receiving low dose colchicine had a significantly lower risk of ischemic CV events compared with patients receiving the placebo, after acute myocardial infarction (MI) and stable coronary artery disease (CAD) [7,8]. The incidence rate of new IS was significantly lower in colchicine users in the COLCOT trial compared with the non-colchicine users [7]. Recent meta-analyses have demonstrated inconclusive but potentially beneficial effects of colchicine on IS prevention. These studies were limited by small sample size and heterogeneous baseline characteristics in the enrolled patients [9,10,11,12,13]. The positive results have mainly come from patients after CAD. Unstable plaque may also contribute to early stroke recurrence in patients with acute IS. However, different subtypes may have diverse underlying mechanisms, which could confound the clinical effects of study drugs. This raises the question of whether recent use of colchicines is associated with fewer stroke recurrence due to its anti-inflammatory effect in patients with acute IS, and our study aimed to answer this question.

## 2. Methods

### 2.1. Data Source and Patient Identification

This retrospective population-based cohort study included patients who were admitted to hospital due to IS between 1 January 2001 and 31 December 2013, as listed in the National Health Insurance Research Database (NHIRD). The first IS admission for each patient was used as the index date if they suffered from multiple IS episodes during the study period. International Classification of Diseases, Ninth Revision, Clinical Modification (ICD-9-CM) codes were used for the registration of all diagnoses [14,15].

**Figure 1:**
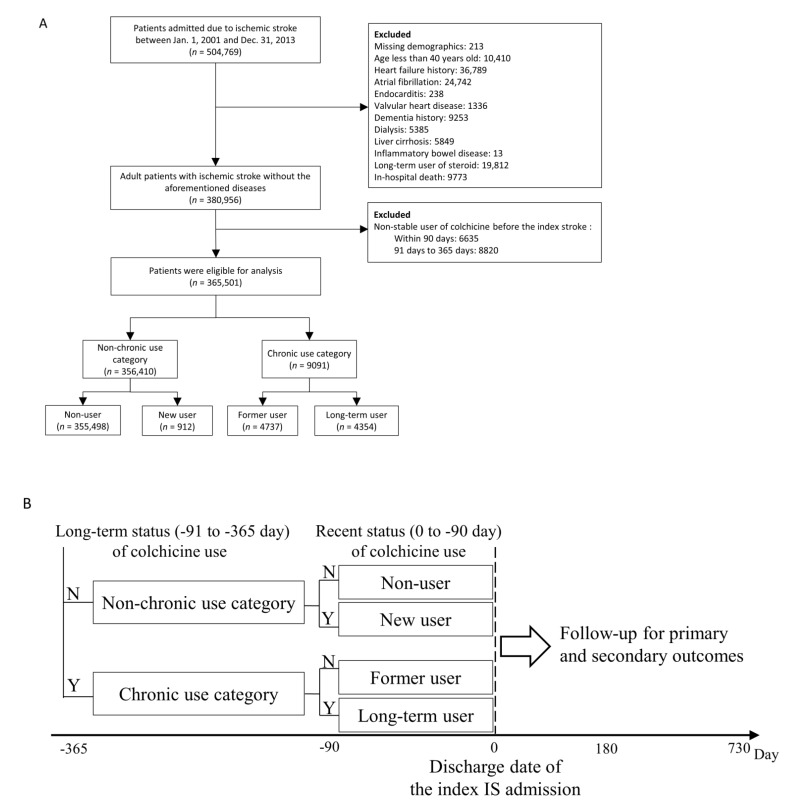
Flowchart of patient selection (**A**), grouping and follow-up and (**B**) within the study (N, no; Y, yes; IS, ischemic stroke).

The patients of interest in the current study were those with a principal discharge diagnosis of IS. Patients who did not have definite cerebral infarction on admission were excluded [16]. The ICD-9-CM diagnostic codes for IS have been validated, and the positive predicted value of principal inpatient diagnoses was 88% [14]. This study focused on non-cardiogenic IS; patients who had possible cardiogenic causes of stroke were excluded. Detailed exclusion criteria are shown in Figure 1. The Ethics Institutional Review Board of our hospital approved the current study, and the need for informed consent was waived as all data was anonymous.

### 2.2. Exposure to the Study Drug

Patients with different statuses of chronic colchicine use could reflect different metabolic and vascular statuses at enrolment. Therefore, the eligible patients were divided into two categories according to their status of chronic colchicine use before the IS index date. The non-chronic use category included patients who did not take any colchicine 91–365 days before the IS index date, whereas the chronic use category included patients who constantly took colchicine 91–365 days before the IS index date. Each category was sub-divided into two groups based on their recent colchicine use (within the last 90 days) before the IS index date. Patients in the non-chronic use category were divided into (1) non-user and (2) new user groups. The non-user group was defined as patients who did not take any colchicine within the 90 days before the IS index date. The new user group included patients who newly received colchicine during this 90-day period. Patients in the chronic use category were divided into (3) former user, and (4) long-term user groups. The former user group included patients who stopped using colchicines within the 90 days before the IS index date. The long-term user group was defined as patients who kept taking colchicine during this 90-day period.

We extracted information on medication use from the claims data for pharmacy refills for chronic illnesses or outpatient visits. We defined patients as stable users if they received a prescription for colchicine for 28 or more days during either the 90 days or 91–365 days before the IS index date. Patients who were prescribed colchicines for less than 28 days in these two periods (non-stable users) were excluded (Figure 1). The definition of drug exposure used in this study was frequently adopted in previous pharmaco-epidemiological studies for the evaluation of drug effectiveness and adverse events [17,18].

### 2.3. Covariates

The patient’s sex and age during their index hospitalization were obtained. The medical records before the index date of hospitalization were traced to track any history of comorbidities. We defined previous stroke and myocardial infarction (MI) history by using any inpatient diagnosis prior to the index date. Underlying comorbidities were defined if the patient had at least 2 outpatient diagnoses or an inpatient diagnosis in the previous year. Most of the diagnostic codes for these events and comorbidities were validated in previous NHIRD studies [19]. We adjusted the average number of antihypertensive drugs and the type of oral antidiabetic drugs to militate the bias associated with different levels of blood pressure and blood sugar levels [20]. We used Charlson Comorbidity Index scores and estimated National Institutes of Health Stroke Scale (NIHSS) to access the patient’s overall systemic health and severity of stroke [21]. The use of medications was captured via the Anatomical Therapeutic Chemical codes, which were defined as at least 2 prescriptions in outpatient visits or any single refill for chronic illness in a pharmacy in the previous 3 months.

### 2.4. Outcome Measurement

The primary outcome in this study was recurrent IS during the 2-year follow up. The secondary outcomes included CV death, all-cause mortality, and new diagnosed AF. Recurrent IS was considered when patients were admitted primarily due to IS during the follow-up period. AF was diagnosed based on the ICD-9-CM code (427.31) in two consecutive outpatient visits or one hospital admission. The positive predictive value of AF diagnosis was around 90% in a previous validation study [22]. The definition of all-cause mortality and CV death were the same as those in the NHIRD registry data [16,23]. We calculated the follow-up period from the day the patient was discharged from their index hospitalization to the day of death, event occurrence, 2 years after the index date or until 31 December 2013, whichever occurred first.

### 2.5. Statistical Analysis

When comparing the risk of outcomes among multiple treatment groups, we conducted an inverse probability of treatment weights (IPTW) for propensity scores (PSs) to balance the baseline characteristics among groups. As there were multiple treatment groups in this study, we estimated the PSs using the generalized boosted model (GBM) [24]. Table 1 lists the variables included in the PS estimation, except that the follow up duration was replaced with the index date. The balance among the multiple treatment groups before and after GBM-IPTW was assessed using standardized differences (STD), in which an absolute value less than 0.2 indicated a small difference between groups [24].

The event rates of outcomes as well as survival analyses were estimated in the cohort after IPTW. The risk of fatal outcomes among treatment groups was compared using the Cox proportional hazard model. The incidence of other time-to-event outcomes among the treatment groups was compared using the Fine and Gray subdistributional hazard model, which considered all-cause mortality as a competing risk. The treatment group was the only one explanatory variable in the aforementioned survival analyses. We further conducted pre-specified subgroup analyses on the primary outcome using the following subgroup variables: age, previous IS, previous MI or CAD, diabetes mellitus, hypertension, CKD, and use of statins. A two-sided *p* value < 0.05 was considered to be statistically significant and no adjustment of multiple testing (multiplicity) was made in this study. Statistical analyses were performed using SAS version 9.4 (SAS Institute, Cary, NC, USA), including the ‘phreg’ procedure for conducting survival analyses and the ‘TWANG’ macro for GBM-IPTW estimation.

## 3. Results

### 3.1. Study Patients

504,769 patients admitted due to IS were available in the NHIRD between 2001 and 2013. We mainly excluded patients who had a history of heart failure (*n* = 36,789), AF (*n* = 24,742), endocarditis (*n* = 238) and valvular heart disease (*n* = 1336). Additionally, 10,410 patients who were less than 40 years old and 9773 patients who died in-hospital were also excluded. In addition, we excluded 15,455 individuals who were non-stable colchicine users before the IS index date. Finally, 365,501 IS patients were confirmed as eligible for inclusion within the study analyses. There were 356,410 patients in the non-chronic use category, including 355,498 in the non-user group and 912 in the new user group. There were 9091 patients in the chronic use category, including 4737 in the former user group and 4354 in the long-term user group (Figure 1A,B).

### 3.2. Baseline Characteristics

Before the GBM-IPTW, patients in the long-term user group were older (70.0 ± 10.8 years old), and had a higher prevalence of gout (84.3%), hypertension (87.6%), previous MI (4.1%), CAD (27.9%), and CKD (11.6%). In addition, patients in the long-term user group had the highest frequency of statin (14.7%), non-steroidal anti-inflammatory drug (48.3%), steroid use (6.1%), multiple antihypertensive drugs (1.73 ± 1.22) and antiplatelets use (aspirin: 27.0%) (Table 1). Conversely, patients in the new user group had the highest CCI score (2.9 ± 1.8). The estimated NIHSS scores were similar among the four groups (maximum absolute standardized difference [MASD] = 0.04). After GBM-IPTW, all baseline characteristics and medications were well balanced among the four groups except for a higher frequency of CAD (29.5%; MASD = 0.26) and aspirin use (27.6%; STD = 0.27) in the long-term user group (Supplemental Table S1).

### 3.3. Primary Outcome

**Figure 2:**
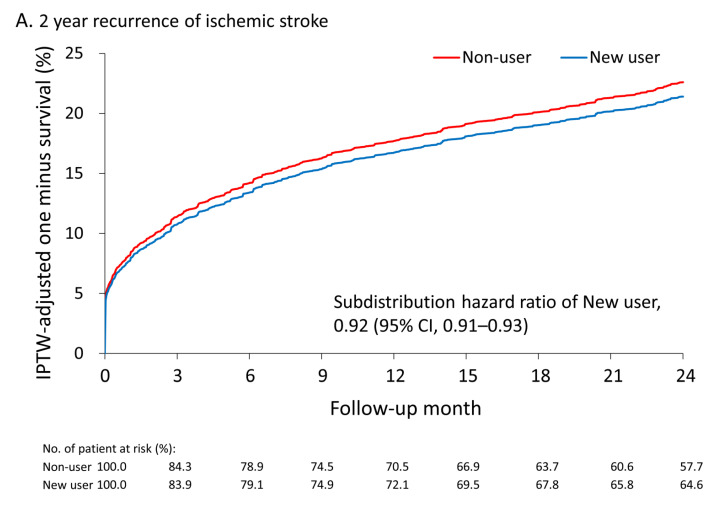

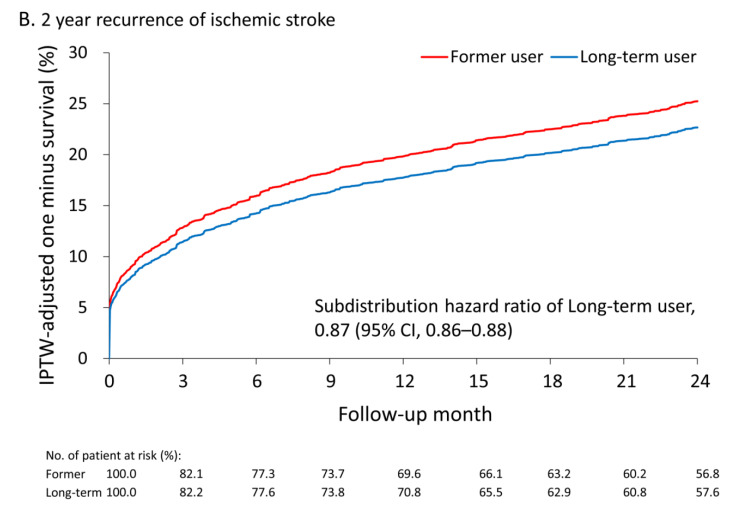
The cumulative incidence of recurrent ischemic stroke during the 2-year follow-up between new colchicine users and non-users (**A**) and between long-term colchicine users and former colchicine users (**B**) in the cohort after IPTW. IPTW, inverse probability of treatment weights.

Long-term outcome of study patients before GBM-IPTW was presented in the supplemental Table S2. After GBM-IPTW, the mean follow-up periods were slightly shorter in the long-term user (4.9 ± 3.4years) and former user (4.9 ± 3.5 years; MASD = 0.21) groups. The primary outcome was compared between the 2 groups in each category. In the non-chronic use category, patients in the new user group had a lower risk of recurrent IS within 6 months compared with the non-user group (subdistribution hazard ratio [SHR], 0.95; 95% confidence interval [CI], 0.94–0.97). In the chronic use category, patients in the long-term user group also had a lower risk of recurrent IS within 6 months compared with the patients in the former user group (SHR, 0.87; 95% CI, 0.86–0.88; Table 2). The advantages of colchicine use on recurrent IS remained in the new user (SHR, 0.92; 95% CI, 0.91–0.93) and long-term user (SHR, 0.87; 95% CI, 0.86–0.88) groups at the 2-year follow up. The cumulative incidence plot shows a lower trend of recurrent IS in the new user and long-term user groups compared with the non-user and former user groups, respectively (Figure 2A,B).

### 3.4. Secondary Outcomes

Compared with the non-user group, the new user group had a higher risk of all-cause mortality (SHR, 1.18; 95% CI, 1.17–1.20), and CV death (SHR, 1.28; 95% CI, 1.26–1.31) at the 2-year follow-up (Table 2). Compared with the former user group, the long-term user group also had a higher risk of all-cause mortality (SHR, 1.10; 95% CI, 1.08–1.12), and CV death (SHR, 1.11; 95% CI, 1.09–1.14) at the 2-year follow-up. The incidence rate of new diagnosed AF was lower in the new user group (SHR, 0.91; 95% CI, 0.88–0.93) but was higher in the long-term user group (SHR, 1.61; 95% CI, 1.57–1.65) at the 2-year follow up when compared with the non-user and former user groups, respectively.

### 3.5. Subgroup Analyses for the Risk of Recurrent IS

**Figure 3:**
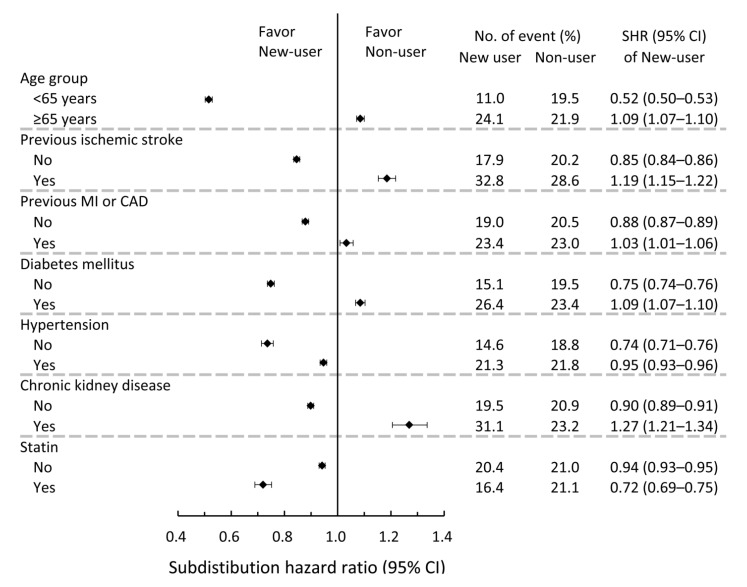
Pre-specified subgroup analyses comparing the risk of 2-year recurrent ischemic stroke between new colchicine users and non-users in the study cohort after IPTW. IPTW, inverse probability of treatment weights.

Subgroup analyses were performed between the new users and non-users. There was a significant difference between colchicine use and a history of IS, CAD or MI, diabetes mellitus, hypertension, and CKD before the index event. The observed effect of colchicine in reducing recurrent IS risks was less apparent in patients who had these underlying diseases. Significant interactions were also observed between colchicine use and concurrent statin use. The observed effect of colchicine in reducing recurrent IS risks was also more profound in patients who concurrently took statins (*p* < 0.05; Figure 3). Subgroup analyses for the chronic use category are shown in Supplemental Figure S1.

## 4. Discussion

Our study showed that colchicines may have a marginal protective effect on IS recurrence in patients with non-cardiogenic IS. Such beneficial effects could be concordantly noted among patients who newly received colchicine and long-term colchicine users who maintained their colchicine use during IS events. This benefit could be observed as soon as 6 months and might last up to 2 years after IS. Although a non-significant IS protective effect of colchicine in patients with chronic CAD was noted in the low-dose colchicine (LoDoCo2) trial [25], our results still reflect a similar trend to the COLCOT study showing that colchicine protected against IS in patients after MI [7], and also support a marginal protective effect of colchicine use for non-cardiogenic IS in Asian patients. We are eager for the results of randomized clinical trials to provide a more conclusive answer.

The main mechanism for colchicine’s vascular protectiveness is its anti-inflammatory effect [3]. Plaque inflammation could be more profound after acute IS and may lead to higher stroke recurrence [26]. Colchicine inhibits the levels of interleukin-6 (IL-6) and C-reactive protein [27], and this could play a role in reducing cardiovascular events after MI [7]. Elevated IL-6 predicts adverse outcomes in patients after acute coronary syndrome [28]; however, the influence of elevated IL-6 on recurrent IS after stroke could be less profound [29,30]. Within the atherosclerotic process, there remain some differences between CAD and carotid artery diseases, including the roles of the inflammatory response [31]. Unlike CAD patients who primarily have atherosclerosis, IS patients are classified into various subgroups according to different underlying cause of their condition. Despite such differences between CAD and IS patients, our results demonstrated that colchicine use had a marginal protective effect in patients with IS. In addition, our subgroup analyses revealed that concurrent use of statins may potentiate the protective effect of colchicine on recurrent IS. Statins have anti-inflammatory effects and help stabilize plaque inflammation, which might strengthen the anti-inflammatory effect of colchicine on IS protection. In addition, colchicine may increase the concentration of statins through CYP 3A4 interactions [32]. Additionally, it should be noted that distribution of IS subtypes is particularly important in Asian populations, due to their higher frequency of lacunar infarction and small vessel disease [33]. Several medications have shown discordant clinical effects between large artery atherosclerosis and other subtypes of IS patients [6,34]. Theoretically, inflammatory responses also play a role in small vessel disease [35,36], and hemorrhagic stroke [37]. However, such responses are much lower than those in cardiogenic and large artery atherosclerosis subtypes [35]. Using the NHIRD, it is difficult to identify IS subtypes. Our results remained insufficient to determine whether IS patients of different subtypes receive equal benefits from colchicine treatment.

Colchicine may have anti-fibrosis effects [27]. In patients after MI, colchicine may help to reduce myocardial fibrosis and improve cardiac hemodynamic parameters [38,39]. Colchicine helps to reduce the occurrence of AF after cardiac interventions [40,41]. Both poor cardiac function and AF are common IS risk factors. Moreover, atrial fibrosis without AF is also a potential source of embolic stroke of undetermined origin [42]. Although the incidence of new IS was significantly decreased in colchicine users after MI, the frequency of newly diagnosed AF was not different between the colchicine and non-colchicine users in the COLCOT study and LoDoCo study [7,25]. In our study, new colchicine users had a significantly lower incidence of AF diagnosis compared with non-users, indicating that the reduction in AF occurrence could be a potential mechanism for the IS reducing effect of colchicine. However, long-term colchicine users in this study did not show a similar protective trend for AF diagnosis when compared with the former users. It is well known that the incidence of AF could be higher in gout patients [43], suggesting that confounding by this indication could still influence our results [44]. Our results suggest that a thorough cardiac rhythm monitoring should be incorporated into the study protocol of clinical trials addressing this issue to clarify the mechanism of colchicine’s IS protective effects. This could also help to better select patients who would benefit from colchicine treatment.

Our study demonstrated that both continuous use of colchicine in former users and a new prescription of colchicine during IS events can have universal benefits. Plaque inflammation can be most profound after IS and could lead to higher stroke recurrence [26]. This could explain why our patients had a protective effect 6 months after IS events. Our results showed higher rates of all-cause mortality and CV death in new and long-term colchicine users. A recent randomized control trial also reported a higher mortality in colchicine users after MI [45], but the colchicine users in this real-world study could be those who have a higher frequency of gout attacks [46], and worse control of metabolic syndromes; CAD and vascular diseases may develop more frequently in this population [44,47,48]. This could be a source of bias to our study results.

The current study had limitations. First, some patients may have had chronic colchicine use because of frequent gouty arthritis rather than inflammatory modulation for CV diseases [46]. We tried our best to mitigate this bias, residual or unmeasured confounders that could have increased the incidence of all-cause mortality, and CV death. The long-term user group had the highest frequency of CAD and aspirin use at baseline. In addition, patients with co-morbidities also had a worse IS protective effect in the subgroup analyses [38], suggesting that confounding by indication could have influenced our secondary outcomes. In such circumstances, the protective effect of colchicine on IS recurrence should be under-estimated. Second, drug adherence may have influenced the study results. The duration of colchicine use may also confound our negative study results. Third, the ICD-9-CM codes could have been incorrectly recorded in the claims database. However, medical reimbursement specialists review and inspect all of the insurance claims so this should minimize this potential error. Fourth, the causal effects of colchicine should be interpreted cautiously as this was an observational study. These exploratory results remain insufficient to provide conclusive answers with a high standard of evidence. Fifth, the statistical analyses could possibly amplify the significance of study findings. Lastly, the generalizability of our conclusions to other ethnicities remains uncertain. Future clinical trials may help to confirm or refute the findings of the current study.

## 5. Conclusions

Our study demonstrated that recent use of colchicines in acute non-cardiogenic IS patients is associated with marginally fewer incidences of recurrent IS in an Asian population. This protective effect was observed at both 6 months and 2 years after IS. Concomitant use of statins with colchicine may potentiate its protective effects. Results of clinical trials are needed to give a more conclusive answer, particularly in the atherosclerotic IS subgroup.

## Figures and Tables

**Table 1:** Baseline characteristics of patients before IPTW.

	Non-Chronic Use Category		Chronic Use Category		
Variable	Non-User (*n* = 355,498)	New User (*n* = 912)	Former User (*n* = 4737)	Long-Term User (*n* = 4354)	MASD
Age, years	68.6 ± 11.8	69.5 ± 11.1	69.6 ± 11.1	70.0 ± 10.8	0.12
Age group, year					
40–64 years	131,323 (36.9)	299 (32.8)	1527 (32.2)	1321 (30.3)	0.14
65–74 years	106,785 (30.0)	290 (31.8)	1553 (32.8)	1451 (33.3)	0.07
≥75 years	117,390 (33.0)	323 (35.4)	1657 (35.0)	1582 (36.3)	0.07
Male sex	202,812 (57.1)	686 (75.2)	3678 (77.6)	3553 (81.6)	0.50
Hospital level					
Medical center (teaching hospital)	102,367 (28.8)	266 (29.2)	1359 (28.7)	1380 (31.7)	0.07
Regional/district hospital	253,131 (71.2)	646 (70.8)	3378 (71.3)	2974 (68.3)	0.07
Comorbidity					
Previous ischemic stroke	34,395 (9.7)	95 (10.4)	454 (9.6)	501 (11.5)	0.07
Previous hemorrhagic stroke	3910 (1.10)	12 (1.32)	45 (0.95)	42 (0.96)	0.04
Gout	18,267 (5.1)	601 (65.9)	3484 (73.5)	3670 (84.3)	3.08
Diabetes mellitus	136,455 (38.4)	344 (37.7)	1688 (35.6)	1489 (34.2)	0.09
Hypertension	265,395 (74.7)	768 (84.2)	4077 (86.1)	3813 (87.6)	0.30
Previous myocardial infarction	7104 (2.0)	30 (3.3)	152 (3.2)	180 (4.1)	0.15
Coronary artery disease	68,294 (19.2)	254 (27.9)	1246 (26.3)	1213 (27.9)	0.22
Chronic kidney disease	9352 (2.6)	95 (10.4)	420 (8.9)	504 (11.6)	0.54
COPD	30,791 (8.7)	130 (14.3)	576 (12.2)	498 (11.4)	0.20
Dyslipidemia	116,734 (32.8)	352 (38.6)	1939 (40.9)	1689 (38.8)	0.17
Previous malignancy	15,448 (4.3)	32 (3.5)	232 (4.9)	191 (4.4)	0.07
Carotid stenting or endarterectomy	654 (0.18)	2 (0.22)	9 (0.19)	16 (0.37)	0.04
CCI score	2.5 ± 1.6	2.9 ± 1.8	2.7 ± 1.8	2.8 ± 1.7	0.27
Estimated NIHSS	6.6 ± 5.1	6.5 ± 5.0	6.7 ± 5.3	6.8 ± 5.3	0.04
Estimated NIHSS group					
≤5	244,478 (68.8)	629 (69.0)	3285 (69.3)	2933 (67.4)	0.04
6–13	68,757 (19.3)	187 (20.5)	869 (18.3)	875 (20.1)	0.06
>13	42,263 (11.9)	96 (10.5)	583 (12.3)	546 (12.5)	0.06
Anti-hypertensive agent					
ACEI/ARB	164,366 (46.2)	498 (54.6)	2504 (52.9)	2381 (54.7)	0.17
CCB	149,514 (42.1)	458 (50.2)	2289 (48.3)	2319 (53.3)	0.23
Alpha-blocker	20,721 (5.8)	82 (9.0)	434 (9.2)	457 (10.5)	0.20
Beta-blocker	87,688 (24.7)	261 (28.6)	1409 (29.7)	1406 (32.3)	0.18
Thiazide	18,483 (5.2)	59 (6.5)	274 (5.8)	235 (5.4)	0.06
Loop diuretics	21,111 (5.9)	92 (10.1)	427 (9.0)	518 (11.9)	0.25
Spironolactone	3462 (1.0)	16 (1.8)	64 (1.4)	66 (1.5)	0.08
Others	7770 (2.2)	31 (3.4)	115 (2.4)	147 (3.4)	0.08
Number of anti-hypertensive agents					
0	103,446 (29.1)	197 (21.6)	1000 (21.1)	776 (17.8)	0.28
1–2	194,957 (54.8)	495 (54.3)	2706 (57.1)	2478 (56.9)	0.06
3–4	54,429 (15.3)	205 (22.5)	978 (20.6)	1027 (23.6)	0.20
≥5	2666 (0.75)	15 (1.64)	53 (1.12)	73 (1.68)	0.08
Average numbers of antihypertensive agents	1.33 ± 1.16	1.64 ± 1.24	1.59 ± 1.20	1.73 ± 1.22	0.34
Antidiabetic agent					
Biguanide (Metformin)	78,222 (22.0)	142 (15.6)	759 (16.0)	622 (14.3)	0.19
TZD	11,565 (3.3)	31 (3.4)	114 (2.4)	110 (2.5)	0.06
Sulfonylurea	80,173 (22.6)	170 (18.6)	826 (17.4)	755 (17.3)	0.13
DPP4i	9095 (2.6)	13 (1.4)	131 (2.8)	82 (1.9)	0.09
Glinide	11,945 (3.4)	41 (4.5)	162 (3.4)	144 (3.3)	0.07
Alpha glucosidase inhibitors	13,758 (3.9)	39 (4.3)	170 (3.6)	160 (3.7)	0.04
Insulin	16,766 (4.7)	44 (4.8)	188 (4.0)	175 (4.0)	0.04
Average number of antidiabetic agents	0.62 ± 1.02	0.53 ± 0.96	0.50 ± 0.93	0.47 ± 0.88	0.16
Other medications					
Aspirin	61,619 (17.3)	237 (26.0)	1012 (21.4)	1176 (27.0)	0.26
Clopidogrel	4424 (1.2)	28 (3.1)	101 (2.1)	137 (3.1)	0.17
Cilostazol	1808 (0.51)	7 (0.77)	45 (0.95)	43 (0.99)	0.07
Statin	28,865 (8.1)	132 (14.5)	591 (12.5)	638 (14.7)	0.24
Fibrate	19,410 (5.5)	92 (10.1)	365 (7.7)	367 (8.4)	0.20
NSAIDs including Cox-2	78,075 (22.0)	367 (40.2)	1881 (39.7)	2105 (48.3)	0.63
Steroid	8541 (2.4)	45 (4.9)	207 (4.4)	265 (6.1)	0.24
Gout medications					
Allopurinol	3692 (1.0)	190 (20.8)	564 (11.9)	1352 (31.1)	2.40
Benzbromarone	6243 (1.8)	343 (37.6)	775 (16.4)	1519 (34.9)	2.33
Sulfinpyrazone	348 (0.10)	15 (1.64)	48 (1.01)	111 (2.55)	0.65
Febuxostat	8 (0.002)	0 (0.000)	5 (0.106)	3 (0.069)	0.16
Follow-up year	5.0 ± 3.6	5.2 ± 3.5	4.5 ± 3.5	5.1 ± 3.5	0.32

IPTW, inverse probability of treatment weighting; MASD, maximum absolute standardized difference; COPD, chronic obstruction pulmonary disease; CCI, Charlson comorbidity index; NIHSS, National Institute of Health Stroke Scale; ACEI, angiotensin-converting enzyme inhibitors; ARB, angiotensin receptor blockers; CCB, calcium channel blocker; TZD, thiazolidinedione; DPP4i, dipeptidyl peptidase-4 inhibitor; NASIDs, non-steroidal anti-inflammatory drug; Data are presented as frequency (percentage) or mean ± standard deviation.

**Table 2:** Long-term outcomes of study patients after IPTW.

	Number of Event (%)				HR or SHR (95% CI)	
	Non-Chronic Use Category		Chronic Use Category			
Variable	Non-User	New User	Former User	Long-Term User	New User vs. Non-User	Long-Term User vs. Former User
6 month						
Primary outcome						
Recurrence of ischemic stroke	13.6	13.2	15.9	14.2	0.95 (0.94–0.97) *	0.87 (0.86–0.88) *
Secondary outcome						
Cardiovascular death	2.7	5.4	2.8	4.5	2.03 (1.97–2.08) *	1.62 (1.57–1.67) *
All-cause mortality	4.9	6.9	5.6	7.5	1.43 (1.40–1.46) *	1.35 (1.32–1.38) *
New-diagnosedatrial fibrillation	3.3	2.4	3.1	4.3	0.73 (0.70–0.75) *	1.39 (1.35–1.44) *
2 year						
Primary outcome						
Recurrence of ischemic stroke	21.0	20.1	23.5	21.0	0.92 (0.91–0.93) *	0.87 (0.86–0.88) *
Secondary outcome						
Cardiovascular death	6.7	8.8	6.9	7.6	1.28 (1.26–1.31) *	1.11 (1.09–1.14) *
All-cause mortality	12.5	15.2	12.1	13.2	1.18 (1.17–1.20) *	1.10 (1.08–1.12) *
New-diagnosedatrial fibrillation	5.1	4.8	4.2	6.7	0.91 (0.88–0.93) *	1.61 (1.57–1.65) *

Data are presented as frequency (percentage); * *p* < 0.05.

## Data Availability

The datasets generated and/or analyzed during the current study are not completely publicly available due to some of our data being limited from being freely accessed due to the IRB regulations of NHIRD data in Taiwan. However, these data are available from the corresponding author on reasonable request.

## Supplementary Materials

The following are available online at https://www.mdpi.com/article/10.3390/jpm11090935/s1. Supplemental Figure S1. Pre-specified subgroup analyses comparing the risk of 2-year recurrent ischemic stroke between long-term colchicine users and former colchicine users in the study cohort after IPTW. Supplemental Table S1. Baseline characteristics of patients after IPTW. Supplemental Table S2. Long-term outcome of study patients before IPTW.
