# Supplementary material for: Colchicine Use and Risks of Stroke Recurrence in Acute Non-Cardiogenic Ischemic Stroke Patients: A Population-Based Cohort Study
Source: J Pers Med. 2021 Sep 19;11(9):935. doi: 10.3390/jpm11090935 (PMC8470154; doi:10.3390/jpm11090935)
Supplement: Supplementary file 1 [file jpm-11-00935-s001.zip › jpm-1370594-SI.pdf]

Supplementary files

Supplemental Figure S1. Pre-specified subgroup analyses comparing the risk of 2-year recurrent ischemic stroke between long-term colchicine users and former colchicine users in the study cohort after IPTW.

IPTW, inverse probability of treatment weights.

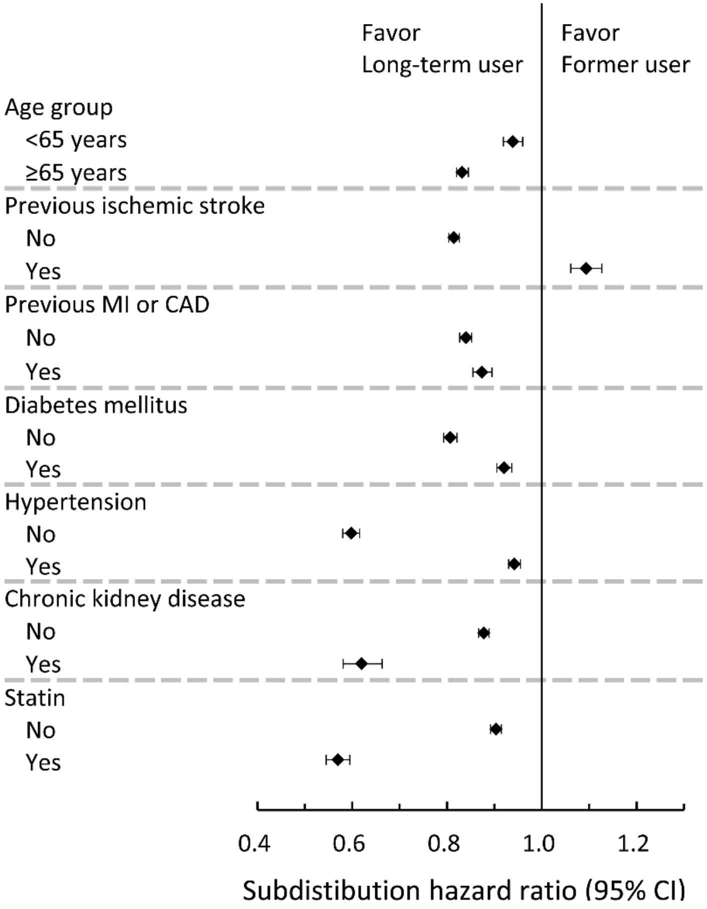

	No. of event (%) Long-term user	No. of event (%) Former user	SHR (95% CI) of Long-term user
**Age group**			
<65 years	20.5	21.8	0.94 (0.92–0.96)
≥65 years	21.2	24.5	0.83 (0.82–0.85)
**Previous ischemic stroke**			
No	19.2	22.9	0.82 (0.80–0.83)
Yes	33.3	28.6	1.09 (1.06–1.13)
**Previous MI or CAD**			
No	19.7	22.7	0.84 (0.83–0.85)
Yes	24.1	26.8	0.87 (0.86–0.90)
**Diabetes mellitus**			
No	18.5	21.8	0.81 (0.79–0.82)
Yes	24.1	26.3	0.92 (0.91–0.94)
**Hypertension**			
No	15.7	23.5	0.60 (0.58–0.62)
Yes	22.3	23.5	0.94 (0.93–0.96)
**Chronic kidney disease**			
No	21	23.2	0.88 (0.87–0.89)
Yes	20.9	31.7	0.62 (0.58–0.66)
**Statin**			
No	21.6	23.5	0.90 (0.89–0.92)
Yes	15.2	23.9	0.57 (0.55–0.60)

Supplemental Table S1. Baseline characteristics of patients after IPTW

Variable	Non-chronic use category		Chronic use category		MASD
	Non-user (*n* = 355,498)	New user (*n* = 912)	Former user (*n* = 4737)	Long-term user (*n* = 4354)	
Age, years	68.6 ± 11.8	69.6 ± 10.3	68.9 ± 11.1	68.9 ± 11.1	0.08
Age group, year					
40-64 years	36.8	30.5	35.0	33.6	0.14
65-74 years	30.1	35.8	31.5	32.6	0.12
≥75 years	33.1	33.7	33.5	33.8	0.02
Male sex	57.6	60.2	61.8	61.9	0.09
Hospital level					
Medical center (teaching hospital)	28.8	26.7	29.6	34.2	0.16
Regional / district hospital	71.2	73.3	70.4	65.8	0.16
Comorbidity					
Previous ischemic stroke	9.7	14.4	10.2	12.8	0.16
Previous hemorrhagic stroke	1.10	0.49	0.76	2.11	0.16
Gout	7.0	9.5	7.9	10.8	0.15
Diabetes mellitus	38.3	43.8	37.9	43.8	0.12
Hypertension	75.0	81.8	77.6	80.0	0.16
Previous myocardial infarction	2.0	2.3	1.9	3.0	0.08
Coronary artery disease	19.4	24.2	19.3	29.5	0.26
Chronic kidney disease	2.8	4.8	3.2	3.1	0.12
COPD	8.8	12.2	10.0	12.7	0.14
Dyslipidemia	33.0	28.4	34.8	36.7	0.18
Previous malignancy	4.3	5.7	4.4	4.9	0.07

Variable	Non-chronic use category		Chronic use category		MASD
	Non-user (*n* = 355,498)	New user (*n* = 912)	Former user (*n* = 4737)	Long-term user (*n* = 4354)	
Carotid stenting or endarterectomy	0.19	0.00	0.07	0.12	0.04
CCI score	2.5 ± 1.6	2.6 ± 1.5	2.4 ± 1.5	2.5 ± 1.4	0.09
Estimated NIHSS	6.6 ± 5.1	5.9 ± 4.3	6.4 ± 4.9	6.1 ± 4.3	0.14
Estimated NIHSS group					
≤5	68.8	75.9	69.9	70.3	0.15
6-13	19.3	15.6	19.6	20.0	0.11
>13	11.9	8.4	10.5	9.8	0.11
Anti-hypertensive agent					
ACEI/ARB	46.4	50.9	48.7	50.9	0.09
CCB	42.3	47.9	46.5	40.9	0.14
Alpha-blocker	5.9	7.2	7.1	7.1	0.05
Beta-blocker	24.8	29.8	25.5	27.5	0.12
Thiazide	5.2	3.6	4.7	5.0	0.07
Loop diuretics	6.0	9.9	6.9	8.3	0.16
Spironolactone	0.98	0.95	0.69	0.34	0.07
Others	2.20	2.99	2.38	0.74	0.15
Number of anti-hypertensive agents					
0	28.8	23.1	26.2	27.9	0.15
1-2	54.9	54.5	56.0	54.7	0.03
3-4	15.5	22.1	17.0	16.9	0.16
≥5	0.77	0.33	0.90	0.47	0.05
Average numbers of antihypertensive agents	1.34 ± 1.16	1.53 ± 1.19	1.42 ± 1.18	1.41 ± 1.17	0.17

Variable	Non-chronic use category		Chronic use category		MASD
	Non-user (n = 355,498)	New user (n = 912)	Former user (n = 4737)	Long-term user (n = 4354)	
Antidiabetic agent					
Biguanide (Metformin)	21.8	23.5	20.3	22.1	0.08
TZD	3.2	3.8	3.0	3.7	0.05
Sulfonylurea	22.4	26.4	20.9	22.3	0.13
DPP4i	2.5	2.4	2.5	2.4	0.01
Glinide	3.4	4.5	4.0	4.7	0.08
Alpha Glucosidase inhibitors	3.9	5.3	2.7	5.6	0.15
Insulin	4.7	4.6	4.8	6.5	0.09
Average number of antidiabetic agents	0.62 ± 1.02	0.71 ± 1.10	0.58 ± 0.99	0.67 ± 1.03	0.12
Other medications					
Aspirin	17.5	21.4	17.2	27.6	0.27
Clopidogrel	1.3	1.1	1.5	2.2	0.10
Cilostazol	0.52	0.78	0.53	0.45	0.05
Statin	8.3	8.9	8.2	10.3	0.08
Fibrate	5.5	6.8	6.0	8.5	0.13
NSAIDs including Cox-2	22.5	29.7	25.6	28.6	0.17
Steroid	2.5	4.5	3.3	2.0	0.16
Gout medications					
Allopurinol	1.5	1.8	1.7	2.3	0.06
Benzbromarone	2.4	3.1	2.7	3.7	0.09
Sulfinpyrazone	0.13	0.09	0.12	0.13	0.01
Febuxostat	0.004	0.000	0.006	0.002	0.01

Variable	Non-chronic use category		Chronic use category		MASD
	Non-user (n = 355,498)	New user (n = 912)	Former user (n = 4737)	Long-term user (n = 4354)	
Follow-up year	5.0 ± 3.6	5.1 ± 3.3	4.9 ± 3.5	4.9 ± 3.4	0.21

IPTW, inverse probability of treatment weighting; MASD, maximum absolute standardized difference; COPD, chronic obstruction pulmonary disease; CCI, Charlson comorbidity index; NIHSS, National Institute of Health Stroke Scale; ACEI, angiotensin-converting enzyme inhibitors; ARB, angiotensin receptor blockers; CCB, calcium channel blocker; TZD, thiazolidinedione; DPP4i, dipeptidyl peptidase-4 inhibitor; NASIDs, non-steroidal anti-inflammatory drug

Data were presented as frequency (percentage) or mean ± standard deviation.

Supplemental table S2. Long-term outcome of study patients before IPTW

Variable	Non-chronic use category		Chronic use category	
	Non-user (*n* = 355,498)	New user (*n* = 912)	Former user (*n* = 4737)	Long-term user (*n* = 4354)
6 month				
Primary outcome				
Recurrence of ischemic stroke	48,135 (13.5)	122 (13.4)	712 (15.0)	642 (14.7)
Secondary outcome				
Cardiovascular death	9,481 (2.7)	35 (3.8)	160 (3.4)	152 (3.5)
All-cause mortality	17,325 (4.9)	53 (5.8)	260 (5.5)	255 (5.9)
New-diagnosed atrial fibrillation	11,716 (3.3)	42 (4.6)	188 (4.0)	164 (3.8)
2 year				
Primary outcome				
Recurrence of ischemic stroke	74,581 (21.0)	210 (23.0)	1,089 (23.0)	996 (22.9)
Secondary outcome				
Cardiovascular death	23,848 (6.7)	79 (8.7)	350 (7.4)	382 (8.8)
All-cause mortality	44,570 (12.5)	129 (14.1)	614 (13.0)	657 (15.1)
New-diagnosed atrial fibrillation	17,970 (5.1)	67 (7.3)	286 (6.0)	265 (6.1)

Data were presented as frequency (percentage).
